# Mind the Map: Technology Shapes the Myeloid Cell Space

**DOI:** 10.3389/fimmu.2019.02287

**Published:** 2019-10-04

**Authors:** Patrick Günther, Joachim L. Schultze

**Affiliations:** ^1^Genomics and Immunoregulation, Life and Medical Sciences Institute (LIMES), University of Bonn, Bonn, Germany; ^2^Platform for Single Cell Genomics and Epigenomics, German Center for Neurodegenerative Diseases and University of Bonn, Bonn, Germany

**Keywords:** monocytes, dendritic cells, human peripheral blood, multidimensional, single-cell RNA sequencing, mass cytometry

## Abstract

The myeloid cell system shows very high plasticity, which is crucial to quickly adapt to changes during an immune response. From the beginning, this high plasticity has made cell type classification within the myeloid cell system difficult. Not surprising, naming schemes have been frequently changed. Recent advancements in multidimensional technologies, including mass cytometry and single-cell RNA sequencing, are challenging our current understanding of cell types, cell subsets, and functional states of cells. Despite the power of these technologies to create new reference maps for the myeloid cell system, it is essential to put these new results into context with previous knowledge that was established over decades. Here we report on earlier attempts of cell type classification in the myeloid cell system, discuss current approaches and their pros and cons, and propose future strategies for cell type classification within the myeloid cell system that can be easily extended to other cell types.

## Introduction

Cell-type identification is an integral part of current immunology (1–5). The immune system as an organ is an assembly of an incredibly complex network of different types of immune cells including T and B lymphocytes, NK cells, innate lymphoid cells, monocytes, macrophages, and dendritic cells (DC), granulocytes including neutrophils, basophils and eosinophils, and mast cells (6). These cell types have specialized roles during homeostasis and infection. Moreover, it became clear that each of these significant immune cell types consists of cell type-specific cell subsets, for example, three monocyte subsets have been described in human peripheral blood, the so-called classical, intermediate, and non-classical monocyte (7). To understand the individual role of each of these subsets, it is crucial to understand the full heterogeneity of these cell types and their subsets to pinpoint the dedicated functions (8). This also needs to be considered in a spatiotemporal fashion, since immune cells are influenced in their function by their respective microenvironment as well as over time (9–11). For example, monocytes accumulate in peripheral reservoirs under homeostatic conditions, but during inflammation, they exert primarily pro-inflammatory effector functions (11–13). At a later time point during the repair phase of an inflammatory response, monocytes are characterized by regulatory properties necessary for tissue repair (14). During the last decade, technological advancements have been used to further refine our understanding of the diversity of cell types and subgroups within the immune system (15). These novel technologies must be put into context with the traditional way of defining cell types mostly relying on low-dimensional data including microscopy, functional assays, and expression of single marker genes. In the first part of the review, we discuss the current principles and strategies of defining cell types and subsets, while highlighting the different aspects of resolving cellular heterogeneity. Here we want to outline how these principles have been applied to the DC/monocyte cell space. Moreover, we will provide a framework for the integration of these recent technological advances to define cell types, subsets, but also functional states of these subsets in an iterative process.

## The Mononuclear Myeloid Cell Space as an Example for Cell Type Definition

Monocytes and DC arise from the myeloid lineage of the hematopoietic system and makeup about 11% of human blood leukocytes (monocytes ~10%, DC ~1%). In humans, monocytes and DC are defined as MHCII^+^CSF-1R^+^ cells, mostly generated through a cascade of continuously differentiating progenitors in the bone marrow. The last shared intermediate is the monocyte-DC progenitor, MDP, which is characterized as a CD45RA^+^CD123^int^CD115^+^ fraction of a heterogeneous granulocyte-monocyte progenitor (GMP) population (16). Using CLEC12A and CD64 expression, a focused monocyte progenitor without DC potential, the common monocyte progenitor (cMoP), was described recently (17). This restricted precursor differentiates via pre-monocytes to monocytes, which in mice egress the bone marrow in a CCR2-dependent fashion (18).

Monopoiesis is highly dependent on the hematopoietic growth factor receptor CSF-1R and is enhanced, especially during infection or “sterile” inflammation (19–22). This phenomenon highlights the function of blood monocytes, which mainly serve as a reservoir for tissue-residing monocyte-derived macrophages and monocyte-derived DCs, especially during inflammation. Under homeostatic conditions, the majority of monocytes are weak phagocytic cells and are less efficient in antigen presentation when compared to DCs and macrophages (14, 23).

Initially described by Steinman and Cohn in the early 1970s DC have been extensively studied in recent decades (24, 25). Nevertheless, the high variability regarding ontogeny, phenotype, tissue localization, and function has hampered to find a comprehensive description of this cell type for a long time. On a functional level, DC are very efficient in phagocytosis and antigen presentation and are therefore crucial for the initiation of an adaptive immune response (23). DC are generated from MDPs giving rise to DC-committed precursor cells called common DC progenitors (CDP) which serve as precursor for plasmacytoid DCs and the two classical DC subtypes cDC1 and cDC2 (26, 27). Recently, a cDC-restricted progenitor cell, the pre-cDC, has been described in mouse and human (5, 28–30). Concerning pDCs, a new model has been recently suggested (1, 31). In fact, 70–90% of pDCs seem to be IRF8-dependent and derive from a different pre-pDC precursor. These cells actively produce type I interferons and do not present antigen very well. Further studies are required to corroborate these recent findings.

## Which Aspects Define Cellular Identity?

### The Traditional Approach: Morphology, Phenotype, and Function

Several characteristics have been used to describe and define cell types and subsets. Initially, morphological characterization by early microscopy and functional observations laid the ground for the idea of different categories of cells. Primarily, features like size, shape of the cell, and/or nucleus, density, and staining behavior for specific dyes were used to separate immune cells into several cell types and subsets (24, 32–37).

Collectively described as mononuclear phagocytic cells, macrophages and monocytes were defined by their unique morphology and ability to take up pathogens and debris (32, 33, 38, 39). Several experiments suggested that blood-derived monocytes will give rise to different types of tissue-resident macrophages, which was comprehended by van Furth and Cohn as the “mononuclear phagocyte system” (MPS) (40). Later, Ralph Steinmann described cells that display a characteristic morphology when cultured on glass surfaces (24). Due to their morphology, he termed them dendritic cells. These DCs were quickly found to be professional antigen presenting phagocytes and were incorporated into the definition of the MPS (25, 41, 42).

The MPS has been defined based on morphology and shared functionality of monocytes, DCs, and macrophages as a broader framework to describe the role of these cell types during homeostasis and immunity. However, the original definition of the MPS cannot adequately explain the heterogeneity of these cell types concerning their origin, tissue localization, disease association, regulation, and function. For example, contrary to the original ideas, blood monocytes are not the only reservoir for tissue-resident macrophages. An enormous body of research established that tissue-resident macrophages are mostly generated by early progenitors during embryogenesis and exhibit to a limited extend the partial ability for self-renewal (43–47). Only some tissues of barrier organs like the intestine rely on the replenishment of tissue-resident macrophages by differentiation of monocytes during adult life, especially during infection or inflammatory conditions (48). Nevertheless, when looking at monocyte-derived and tissue-resident macrophages, we must acknowledge that these cells have a high phenotypic and functional similarity. This redundancy is essential for the (functional) replacement of yolk-sac derived tissue-resident macrophages in some tissues but makes it difficult to find a unified classification.

The use of surface marker detection by monoclonal antibodies and flow cytometry has revolutionized the way of cell type definition throughout immunology. While a functional heterogeneity of monocytes was suggested by several earlier studies (34–37, 49), it was two-color flow cytometry that provided a tool to clearly define two major monocyte subsets by their expression of CD14 and CD16 (50, 51). About 80 to 90% percent of peripheral blood monocytes express CD14 but lack the expression of the Fcγ-receptor III (FcγRIII/CD16). This subset is characterized by a higher phagocytic activity compared to the minor subset expressing CD16 and intermediate levels of CD14. Also, CD16^+^ monocytes can be further separated based on their expression of CD14 into CD14^dim^ CD16^+^ population and a less frequent CD14^+^ CD16^+^ subset (52, 53). The CD14^+^CD16^−^ subset of monocytes is referred to as classical monocytes, monocytes expressing CD14 and CD16 as intermediate monocytes and non-classical monocytes are defined as the CD14^dim^CD16^+^ subset (7, 52, 53). Furthermore, during the last decade, several markers have been suggested for defining the monocyte cell heterogeneity, including Slan and CD2/FcεRI (54–56). However, these markers do not reach the specificity that would be required for an unambiguous definition of cell types or cell subsets (also see below and Box 1).

Box 1 **Proposed framework for the definition of cell types, cell subsets, and functional states of cell types and subsets.**

Cell type definition based on a single parameter space (e.g. only ontogeny) will be inferior to integrated approaches utilizing additional information (ontogeny, -omics data, phenotypic, and functional data). Nevertheless, even with such a large heterogeneous parameter space at hand, cell type definition is still not trivial. We propose a framework to define cell types and their subsets that is based on knowledge from decades of developmental and cell biology, further substantiated with recent developments and results in the field of single-cell omics (165–168). Certainly, such proposal will require larger community involvement and is mentioned as a starting point for discussion. This principle can be extended to define other cell types as well.

According to this framework, “**cell types**” would be defined as follows:

“Cell types” constitute the highest category. Cell types are defined by the lack of transdifferentiation capacity in more than 95% of all physiological and non-physiological conditions. Furthermore, cell types exhibit certain phenotypic, functional and genome-wide (transcriptome, epigenome, other) characteristics that are unique to all cells of a particular type. For immune cells that are terminally differentiated, cell types would include T and B lymphocytes, NK cells, monocytes, macrophages and DC, neutrophils, basophils and eosinophils, mast cells and innate lymphoid cells. For the stem cell and precursor compartment, the hematopoietic stem cell would be one cell type, while all precursors could be another cell type. Particularly in the precursor space, more research is required to define whether—based on this definition—further cell types or only cell subsets (see below) exist. This is similarly true for cell type development during embryogenesis. However, such a framework would certainly guide future research, specifically exploiting experimental systems that would allow answering the question, whether a cell is still capable of transdifferentiating toward another cell type.

“**Cell subsets**” would be defined as follows:

“Cell subsets” are a secondary category within any given cell type. Cell subsets share certain phenotypic, genome-wide (transcriptome, epigenome) and functional features within a given cell type, but are distinct in other phenotypic, functional, or genome-wide features that are unique to them within a cell type. In an ideal setting, these features should not overlap with those features that characterize the cell type. Furthermore, the feature set characterizing a cell subset should not change if cells are analyzed from different compartments (tissues, organs) and under differing conditions (homeostasis, acute inflammation, repair conditions, etc.). Cell subsets can be further distinguished from cell types in that cell subsets can change into another subset of the same cell type to the degree that is higher than 5%. For example, it is known that classical monocytes can further differentiate into non-classical monocytes via the intermediate monocyte subset.

“**Functional states**” are defined as follows:

“Functional states” are the overall current program of any given cell. Again, “functional states” would be defined by a specific pattern of phenotypic, functional and genome-wide characteristics, which ideally would exclude features characterizing cell types or subsets. “Functional states” rely on spatiotemporal information (e.g., location, the cell's individual age, the age of the organism), the activation state (homeostasis, acute, chronic inflammation, repair phase, etc.) and any combination thereof. Clearly, “functional states” can only be defined by integrated approaches and patterns or signatures of many parameters. Single parameter definitions for functional states are very unlikely. Any given cell can be described by combinations of “functional states.” In other words, “functional states” can be linked to intracellular biological modules responsible for different cellular functions. A cell could express pro-inflammatory cytokines and have elevated migratory capacity. “Functional states” can even be shared among different cell types and cell subsets. However, together with the definition of the cell type and subset, a cell can be defined unambiguously according to the three levels of cell type classification.

“Cell types,” “cell subsets,” and “functional states” will be governed by transcriptional programs that are linked to defined and specific networks of transcription factors (TFs) not only single TFs. Therefore, the description of such networks might be another means of defining cells accordingly.

The introduction of functional states will reduce the excessive introduction of new cell types or subsets and—in our view—also represents the well-known plasticity of the myeloid cell space better.

Like monocytes DC have been first described on the basis of their morphological and functional aspects. Here, pDCs are characterized as main type-I interferon (IFN-α/β) secreting cells with plasma cell-like morphology (57). Activation and secretion of type-I interferons are facilitated by recognition of virus-derived nucleic acids, especially by endosomal nucleic acid-sensing Toll-like receptors (TLRs) TLR7 and TLR9 (57). Initially, these cells were identified by several groups under different names, including natural interferon-producing cells, plasmacytoid monocytes, and plasmacytoid T-cells (58–61). Finally, a consensus name, the plasmacytoid DC was introduced and phenotypic markers were defined including human blood dendritic cell antigen (BDCA)-2, human IL-3Rα (CD123) and BDCA-4 (57, 62–65). However, as already mentioned before and described in more detail later, previously reported experiments suggest that this consensus is once again challenged (1, 31) strongly arguing for an iterative process of cell type definition continuously including new information.

Besides pDCs, there are two subsets of myeloid or classical DC (mDC/cDC) that can be distinguished in the Lin–MHC-II+CD11c+ fraction (66, 67) by using the non-overlapping markers CD1c (BDCA1) or CD141 (BDCA3) in flow cytometry (64, 65). These DC subsets have been termed cDC1 (CD141^+^ DC) and cDC2 (CD1c^+^ DCs), respectively, which have been reviewed extensively elsewhere (68–73). While these classical markers are widely used, further markers have also been suggested for subset classification of DCs (73, 74). For instance, CD141^+^ cDC1 can be identified by using antibodies against XCR1 (75, 76), CLEC9A (77–79) and CADM1 (80). Interestingly, all DC populations vary regarding their expression of the pattern recognition receptor family toll-like receptors, which is highly correlated with the functional roles these cells play in T-cell activation. For example, human CD141^+^cDC1 cells express high amounts of TLR3 (81), a pattern recognition receptor highly associated with cross-presentation (82) and thus cDC1s are specialized in presenting intracellular antigens to CD8^+^ T-cells in human and mice (83).

The most abundant subset of blood DCs are CD1c^+^ cDC2s, which can be defined analytically by expression of CD11c, CD1c (BDCA1), and FCεRIa (54, 64, 84). Furthermore, CD1c^+^ cDC2 express high levels of class II MHC molecules like HLA-DR, HLA-DQ, and show a high endocytic capacity, which specializes this DC type for the presentation of exogenous antigen to CD4^+^ T cells (64, 84). As we will outline below, future work will require community efforts to integrate the differential usage of cell subset classification markers to generate consensus nomenclatures.

Collectively, the definition of cell types of the MPS and their subsets was initially based on cellular morphology, further developed by introducing immunophenotyping using antibodies against the respective cell surface markers and complemented by a functional assessment of the cell subsets identified. We spare the many controversial findings throughout this period, which only reflects the limitations of these approaches to generate a widely accepted nomenclature of cell types and subsets.

### Ontogeny as a Concept for Cell Type Definition

A group of leading experts in the field of monocyte, DC, and macrophage biology has recently proposed a nomenclature, which is based mainly on the ontogeny and tissue localization of cells (73). The proposed two-level model defines a cell type, first by its origin (level 1), which is further improved by adding a functional, phenotypic or location information (level 2) of the particular cell type. This aspect of cell type classification and the ontogeny of DCs and monocytes have been reviewed extensively (48, 72, 85).

The usage of cellular origin for cell type classification is beneficial since such approach already segregates distinct, functional units. For example, it was suggested that all phagocytes that are generated by yolk-sac derived progenitors should be referred to as macrophages and cells derived from the hematopoietic lineage as monocyte-derived cells (8, 68). A further advantage of using origin and development of immune cells as a guiding principle for cell type definition is the conservation of ontogeny across species. However, although there is a substantial overlap of ontogenies in human and murine macrophage, monocyte and DC development, there is also considerable disagreement (16, 83, 86–88). Additionally, the ontogeny of myeloid cells is difficult to study in humans, and most results are obtained by mice experiments and then projected to human myeloid cells. Clearly, the ontogeny approach is a very important aspect of cell type definition, but it needs to be combined with other characteristics of cells.

## High-dimensional Approaches Shape the Myeloid Cell Space

Here, we introduce the latest technological advancements that have made substantial contributions to clarify the monocyte/DC compartment. Furthermore, we want to discuss open questions and challenges associated with these new technologies. Multi-dimensional approaches have significantly improved our understanding of the myeloid cell space by providing more features resulting in higher resolution for cell typing. To contextualize this, we want to provide examples that outline how high-dimensional methods have shaped our understanding of heterogeneity in human blood-derived monocytes and DC.

Although conventional flow cytometry has revolutionized cell type classification, it is limited in the number of parameters (markers <20) being analyzed at the same time. In the early 2000s, there were a couple of technological advancements that paved the way to the development of mass cytometry enabling parallel analysis of up to 40 parameters (89–93). This higher depth of data simultaneously enabled a multitude of possibilities for immunological and biomedical sciences, including the high-dimensional assessment of cross-patient cell type dynamics during acute myeloid leukemia (94–97). More recently, multi-color flow cytometry (MCFC) has been introduced, increasing the parameter space to a similar range, as seen in mass cytometry. However, although mass cytometry and MCFC allow high-throughput protein profiling of thousands of cells, the restriction to <40 protein markers may be underrepresenting the true number of variables that are necessary to define the heterogeneity in highly complex biological samples. Besides, these markers have to be selected *a priori*, which may put a bias on the results obtained by mass spectrometry or MCFC. Another revolution was introduced by the development of high-throughput gene expression profiling methods like microarray-based technologies and RNA-sequencing enabling to profile thousands of genes in a single sample (98, 99). This second genomic revolution enables the genome-wide assessment of gene expression, which not only allows to characterize cellular subsets but also to investigate regulatory networks (20, 100–102).

One of the first studies that performed microarray analysis of human DCs compared the transcriptomes of sorted cDC1, cDC2, and pDCs populations from peripheral blood and tonsils to deeply characterize these subsets (103). Robbins et al. performed a comparative study to put the transcriptome data of DC subsets into context of other myeloid and lymphocyte populations in blood (104), which resulted in the identification of important conserved signature genes, thereby strengthening cDC1, cDC2, and pDC as distinct DC subsets. Moreover, assessing transcriptomic data of both murine and human immune cells allowed to align DC subsets across species (104, 105). Another important study performed transcriptome profiling of human blood CD14 and CD16 monocyte populations, three DC subsets pDC, cDC1, and cDC2 as well as their skin counterparts cDC1, cDC2, and skin derived CD14^+^ cells (80).

Notably, cell types like skin cDC1 and cDC2 grouped together with their counterparts isolated from blood, suggesting a high similarity of DC subsets independent from the microenvironment. We extended these findings to compare different DC subsets in many individuals and different tissues [lymphohematopoietic (blood, thymus, spleen) and non-lymphohematopoietic (skin, lung)] allowing to characterize the impact of the microenvironment on the identity of a cell type (74). Integration of immune phenotyping, gene expression profiling, and bioinformatic analysis revealed that DC subsets from blood, spleen, and thymus were transcriptionally conserved, with only minor transcriptomic differences between the same DC subsets across tissues. In contrast, the transcriptomic consequence of the respective microenvironment was stronger in lung and skin subsets. This suggests a higher tissue imprinting of non-lymphohematopoietic DC subsets in barrier organs like lung and skin, when comparing to the tissue imprinting that has been reported for tissue-resident macrophage subsets (47, 100, 106, 107). However, the difference between different DC subsets (cDC1 vs. cDC2) is still larger than the differences between the same DC subset among different tissues (e.g., skin cDC1 vs. blood cDC1).

Collectively, gene expression profiling and comparative bioinformatic analysis have substantially contributed to understand the complex DC networks across species further improving current descriptions of unified and more unbiased classifications (73, 105, 108).

Early transcriptomic approaches of human and mouse monocyte subsets not only helped to deeply characterize these cell types but also presented a framework to validate high conservation of gene expression profiles between mouse and humans (104, 109). For example, a combination of well-designed functional assays and gene expression profiling helped to refine the role of non-classical monocytes as the counterpart to murine “patrolling” Gr1^−^ monocytes (110). Other studies sharpened the definition of the intermediate and non-classical monocytes as distinct cell subset (110–112). Interestingly, these studies revealed a high similarity of non-classical and intermediate monocytes, underlining the transitional nature of these cells, as they show intermediate expression for most of the marker genes differentially expressed between classical and non-classical monocytes. Interestingly, a unique module of class-II MHC genes was highest expressed in the intermediate monocyte population (111).

Measuring RNA rather than protein levels represents one of the major limitations of gene expression profiling methods. While the overall correlation of transcriptome and proteome is relatively high (113, 114), RNA-seq and microarrays do not allow to assess post-translational modifications, which represent a central part of cellular regulation (115, 116). To overcome this limitation, mass cytometry has been utilized to profile post-translational modifications like phosphorylation, methylation, and glycosylation (117, 118). A good example of the value of methods with larger feature size compared to single or few marker studies is the definition of cells expressing the carbohydrate modification 6-Sulfo LacNAc (*Slan*) on the PSGL1 protein. Indeed, myeloid cells presenting Slan initially were termed “SlanDCs” (119–121), while others described an overlap of Slan^+^ cells with non-classical monocytes (122, 123). However, all these studies largely rely on low-dimensional marker assessment by flow cytometry and are not always directly comparable due to differences in their choice of markers or gating strategies. To investigate this in a more unbiased fashion, Roussel et al. defined a 38-marker panel to study human myeloid cells from peripheral blood by mass cytometry (124). A semi-supervised analysis of the data resulted in the identification of distinct monocyte populations, two subsets overlap with markers from classical and intermediate monocytes while there are two subsets of monocytes that are similar to non-classical monocytes. The multi-dimensional analysis maps Slan^+^ cells to the non-classical monocytes and does not show alignment with any DC population. In this study, Slan separates the non-classical monocytes into a Slan^high^ and a Slan^low^ CD14^dim^CD16^+^ population. However, earlier genomic comparisons of sorted Slan^high^ vs. Slan^low^ subsets did not reveal a significant difference between those two populations (110). More recently, by combining index sorting and high-content single-cell RNA-sequencing, we show further evidence that Slan expression does not reflect different cell subsets as the underlying overall transcriptional program is not different between Slan^high^ and Slan^low^ cells. Moreover, we clearly show that Slan+ cells are all non-classical monocytes (125).

Manual gating of monocytes by CD14 and CD16 is biased by the investigator, which is a disadvantage for large multi-center clinical studies. Unsupervised and semi-supervised computational analyses improve the accuracy and reproducibility of subset definitions (95, 117, 124, 126–128). However, interpretation of these results must be performed with special care, since the primary analysis is still dependent on manual parameter settings by the investigator. For example, in contrast to an earlier study utilizing mass cytometry (124) similar profiling of human mononuclear myeloid cells revealed three subsets of human monocytes in two other studies, while others report significant heterogeneity including three non-classical, one intermediate and four classical subsets (22). Interestingly, Hamers et al. identified a non-classical population, which is quite different to other non-classical populations and expresses CD9^+^ CD41^+^ and CD61^+^, which may represent an eosinophil/basophil contamination (129–131). Another interesting observation is the rather low inter-individual difference of human monocyte populations during homeostasis when assessed by mass cytometry (22, 132).

High-throughput gene expression profiling by microarray or RNA-seq has paved the way to understand the regulatory networks within human monocytes and DC. These technologies are indispensable for high-depth characterization of immune cell types. Nevertheless, these population-based methods are not designed to detect further cellular heterogeneity within a sample. The gene expression measurement in a population-based RNA-seq represents an average signal of typically more than 10,000 individual cells, resulting in leveling out any further heterogeneity. Frequently, samples are generated by flow cytometry assisted cell sorting, which relies on the information of a limited set of marker genes. However, if these markers are not sufficient for detecting the full heterogeneity of the tissue, the results may be underestimating the true heterogeneity.

Transcriptional profiling of individual cells by single-cell RNA-seq has been introduced in 2009 (133, 134) and has revolutionized cell type discovery in all fields of biology (135–142), therefore it may be claimed as “third genomic revolution.” Single-cell RNA sequencing approaches allow transcriptional profiling of 10,000s of individual cells. In contrast to population-based RNA-seq, the groups of cells are not defined *a priori*, rather the cell classification is based on the similarity of gene expression profiles.

A series of studies applied single-cell RNA-seq to understand the heterogeneity of human blood DCs and DC progenitors (5, 30, 143). See et al., as well as Villani et al., detected and characterized the conventional subsets, including cDC1, cDC2, and pDC. Surprisingly, beyond these similarities the results differed significantly, strongly arguing that such high-dimensional data require particular care when assigning cell types and cell subsets. We defined cell types and subsets by a combination of function, phenotype and transcriptional profile, which lead to the identification of precursors (pre-cDCs) for the cDC1 and cDC2 subsets in addition to the three main DC subsets (5). To reconcile these two major initial reports, we developed a strategy that allows developing cell type classification consensus based on phenotypic and transcriptional features also including prior knowledge (125). This approach revealed that (1) the AXL^+^Siglec6^+^ DCs (AS-DCs) described by Villani et al. are mainly pre-cDCs as described in (5), (2) Mono4 are contaminating CD56^dim^ NK cells, and (3) cells introduced as CD16^+^ CSF1-R^+^ CTSS^+^ DCs are not belonging to the DC lineage. This general strategy is not restricted to myeloid cells but can be applied to any cell type classification problem in any species (125).

Recently, single-cell RNA-seq has also been used for improving our knowledge about the generation of DCs from bone marrow-derived progenitors. There is evidence that there is much higher flexibility in the development of DC and monocytes than already appreciated. Hematopoietic models that are not based on repeating rounds of division and differentiation (72, 144, 145) allow for incorporation of recent findings that suggest that cDCs can be generated by lymphoid progenitors (146). Also, the latest reports show important evidence that the large majority of pDCs arise from lymphoid progenitors rather than CDPs (1, 31). Probably, a community effort to clarify future naming and nomenclature of these cells is now warranted. Importantly, the recent high-dimensional characterization of pDCs (5, 125, 132, 143) and new insights into their ontogeny in mice (1) could form the basis for such new discussions.

Clearly, this is only the beginning of applying these technologies to open questions concerning the plasticity of the myeloid cell compartment. We also recognize that single-cell RNA-seq data are currently challenging our view on cell type classification and function within the myeloid cell compartment. However, in the long run, we are convinced that the higher information content per cell will give us a much better understanding of individual cells within any given tissue, organ, or inflammatory response.

## Proposal of General Principles for Cell Type Definitions

Considering the apparent ease, with which different cell types were characterized based on morphological differences a century ago (39), our capabilities to simultaneously measure hundreds to thousands of parameters per single cell seem to decrease our ability to agree on defined cell types and cell subsets (1, 5, 31, 143). The ability to detect heterogeneity between individual cells has extended to biological differences that are not related to questions concerning cell type or cell subset. The best-characterized biological process in single cell –omics data being cell cycle in proliferating cells (147–149). Certainly, cell cycle differences should not classify two cells of the same type as different cell types or subsets. Stochastic behaviors of single cells, e.g., in transcription (150, 151) would be another biological phenomenon that should not impact on cell classification aspects. Furthermore, data sparsity, still very apparent in all sequencing-based single cell technologies, requires attention, when dealing with cell type definitions.

Similarly, important is the question, whether all biased approaches requiring feature selection (e.g., which markers to be analyzed) prior to analysis are good starting points for cell type definitions. These would include all multi-color flow cytometry and single-cell mass spectrometry approaches. Potentially a more appropriate approach would be the combination of markers (chosen by the investigator) with unbiased approaches provided by single cell sequencing-based technologies. This is crucial since it allows to link the enormous body of research that has been performed with flow cytometry-defined cell populations (e.g. ontogeny) with results obtained by analysis of high-dimensional data. For example, index sorting based on previously defined cell surface markers combined with scRNA-seq might be a better way of defining the cell population structure as well as the practicality of certain protein markers to capture the population structure (125, 152, 153). Alternative but significantly more expensive approaches are based on the combination of full transcriptome scRNA-seq and oligonucleotide-labeled antibodies (154, 155). It can be expected that these approaches require iterations of experiments until markers are identified that truly reflect the underlying population structure. In this context, it is important to note that even such large endeavors such as the Human Cell Atlas will require the integration of additional layers of information in addition to scRNA-seq data. Furthermore, we postulate that these iterations will lead to consensus maps as a basis for cell type definitions (125). Very much like the cluster of differentiation (CD) workshops for antibodies (156), a community effort will be necessary to agree on the different versions of such consensus maps of individual cell types.

However, even if the combination of truly unbiased single cell –omics approaches and antibody-based techniques leads to novel consensus maps of immune cells including the myeloid cell space, we propose that each cell type and more importantly each cell subset requires to be functionally characterized, as we have previously demonstrated for human DCs in blood (5). In other words, we strongly argue that a final definition of a cell subset should be validated on functional differences and not only on transcriptional and phenotypic differences.

Once cell types are defined under homeostatic conditions, which is a major goal of the Human Cell Atlas (157), an even more daunting task will be to define cell types and subsets under pathophysiological processes. While certain cell types will be under developmental trajectories (cell states) under physiological conditions, the space for different cell states in disease settings will further increase (158). More importantly, under these conditions, there will be mainly changes in parameters related to biological function rather than features defining cell types or subset. A major goal for further cell type definitions will be to integrate these functional states and trajectories. In this context, we propose cell types as the highest level to distinguish cells. For example, DC, monocytes, and macrophages would qualify as individual cell types, while pDC, cDC1, and cDC2 would qualify as DC subsets (5, 125). Each of these subsets can exist in different functional states that depend on location, differentiation stage, acute or chronic activation signals, to name only a few (69, 74). Again, even for functional states, we would propose to define cells based on hundreds of parameters measured by single cell –omics technologies to be combined with classical marker strategies but finally also integrate functional readouts for these cellular states.

Even if we can agree on such an approach, the question remains, how this can be realized technically? In fact, this is not a mere technical question, as it requires to consider methods that are more independent of investigator bias. For example, we strongly suggest building approaches that will allow us to build cell type definitions based on machine learning rather than on investigator-driven and individualized analysis pipelines. Single-cell transcriptomics algorithms as they are implemented in singleR (159) or scMatch (160) are good starting points. Nevertheless, they still heavily rely on an investigator's interpretation of such high-feature data spaces. Cell type definition could be a classification problem requiring the respective machine learning as they are used for classifier generation in other areas (161, 162). We do not favor solely data-driven machine learning but would suggest the integration of prior knowledge. First attempts to develop such methods are currently underway, and we will soon know, whether the introduction of machine learning based cell class prediction will truly aid our attempts to make sense of the hundreds to thousands of parameters that we now can routinely measure from single cells.

## Summary and Outlook

Since the discovery of myeloid cells more than a century ago, we have learned a lot about these important immune cells. Their enormous plasticity is fascinating and challenging at the same time. Not surprisingly, cell type definitions and nomenclature—up to the day—have been changed or updated regularly (48, 68, 108, 163, 164). A unified nomenclature is the basis for an effective communication among scientists and will accelerate discovery of novel therapeutics. Moreover, high-dimensional profiling of samples will facilitate to compare results and cell types across experiments, tissues and species. Even with the highest number of parameters known per any given cell, we still differ in our interpretations of certain cell types within the myeloid compartment. While it will be rather critical to include prior knowledge when labeling cells based on high-dimensional single cell data, we need to develop better tools based on robust mathematical rules that help us to determine cellular phenotypes and functions less ambiguously. With the emergence of powerful machine learning and AI-based methodology, the time has probably come to utilize such approaches to our benefit when describing cell types, cell subsets, and their functional states. Irrespective of the power of such approaches, we also need to accept that we are far from a complete understanding of these cells. Additional layers of information, for example, epigenetic information, will have to be included in cell type definitions as they arise. Therefore, we foresee numerous iterations of defining cell types and their functions in the decades to come. In other words, consensus maps of cell types and subsets that we agree on today will form the basis for newer maps with updated information content in the future. A potential framework for such a community-based effort has been outlined here.

## Author Contributions

PG and JS conceived and wrote the manuscript.

### Conflict of Interest

The authors declare that the research was conducted in the absence of any commercial or financial relationships that could be construed as a potential conflict of interest.

## References

[1] Dress RJ Dutertre C-A Giladi A Schlitzer A Low I Shadan NB. Plasmacytoid dendritic cells develop from Ly6D+ lymphoid progenitors distinct from the myeloid lineage. Nat Immunol. (2019) 20:852–64. 10.1038/s41590-019-0420-3 31213723

[2] Van Galen P Hovestadt V Wadsworth MH Aster JC Lane AA Bernstein Correspondence BE. Single-Cell RNA-Seq reveals AML hierarchies relevant to disease progression and immunity. Cell. (2019) 176:1265–81.e24. 10.1016/j.cell.2019.01.031 30827681 PMC6515904

[3] Thorsson V Gibbs DL Brown SD Wolf D Bortone DS Ou Yang T-H. The immune landscape of cancer. Immunity. (2018) 48:812–30.e14. 10.1016/j.immuni.2018.03.023 29628290 PMC5982584

[4] Ibarra-Soria X Jawaid W Pijuan-Sala B Ladopoulos V Scialdone A Jörg DJ. Defining murine organogenesis at single-cell resolution reveals a role for the leukotriene pathway in regulating blood progenitor formation. Nat Cell Biol. (2018) 20:127–34. 10.1038/s41556-017-0013-z 29311656 PMC5787369

[5] See P Dutertre CA Chen J Günther P McGovern N Irac SE. Mapping the human DC lineage through the integration of high-dimensional techniques. Science. (2017) 356:eaag3009. 10.1126/science.aag3009 28473638 PMC7611082

[6] Parkin J Cohen B. An overview of the immune system. Lancet. (2001) 357:1777–89. 10.1016/S0140-6736(00)04904-7 11403834

[7] Ziegler-Heitbrock L Ancuta P Crowe S Dalod M Grau V Hart DNDN. Nomenclature of monocytes and dendritic cells in blood. Blood. (2010) 116:e74–80. 10.1182/blood-2010-02-258558 20628149

[8] Guilliams M Mildner A Yona S. Developmental and functional heterogeneity of monocytes. Immunity. (2018) 49:595–613. 10.1016/j.immuni.2018.10.005 30332628

[9] Garcia-Bonilla L Faraco G Moore J Murphy M Racchumi G Srinivasan J. Spatio-temporal profile, phenotypic diversity, and fate of recruited monocytes into the post-ischemic brain. J Neuroinflamm. (2016) 13:285. 10.1186/s12974-016-0750-0 27814740 PMC5097435

[10] Kepler TBB Chan C. Spatiotemporal programming of a simple inflammatory process. Immunol Rev. (2007) 216:153–63. 10.1111/j.1600-065X.2007.00500.x 17367341

[11] Qi H Kastenmüller W Germain RNN. Spatiotemporal basis of innate and adaptive immunity in secondary lymphoid tissue. Annu Rev Cell Dev Biol. (2014) 30:141–67. 10.1146/annurev-cellbio-100913-013254 25150013

[12] Vento-Tormo R Efremova M Botting RA Turco MY Vento-Tormo M Meyer KB. Single-cell reconstruction of the early maternal–fetal interface in humans. Nature. (2018) 563:347–53. 10.1038/s41586-018-0698-6 30429548 PMC7612850

[13] Schultze JL. Chromatin remodeling in monocyte and macrophage activation. Adv Protein Chem Struct Biol. (2017) 106:1–15. 10.1016/bs.apcsb.2016.09.001 28057208

[14] Geissmann F Manz MG Jung S Sieweke MH Merad M Ley K. Development of monocytes, macrophages, and dendritic cells. Science. (2010) 327:656–61. 10.1126/science.1178331 20133564 PMC2887389

[15] Neu KEE Tang Q Wilson PCC Khan AAA. Single-cell genomics: approaches and utility in immunology. Trends Immunol. (2017) 38:140–9. 10.1016/j.it.2016.12.001 28094102 PMC5479322

[16] Lee J Breton G Oliveira TYK Zhou YJ Aljoufi A Puhr S. Restricted dendritic cell and monocyte progenitors in human cord blood and bone marrow. J Exp Med. (2015) 212:385–99. 10.1084/jem.20141442 25687283 PMC4354373

[17] Kawamura S Onai N Miya F Sato T Tsunoda T Kurabayashi K. Identification of a human clonogenic progenitor with strict monocyte differentiation potential: a counterpart of mouse cMoPs. Immunity. (2017) 46:835–48.e4. 10.1016/j.immuni.2017.04.019 28514689

[18] Jacquelin S Licata F Dorgham K Hermand P Poupel L Guyon E. CX3CR1 reduces Ly6Chigh-monocyte motility within and release from the bone marrow after chemotherapy in mice. Blood. (2013) 122:674–83. 10.1182/blood-2013-01-480749 23775714

[19] Patel AA Zhang Y Fullerton JN Boelen L Rongvaux A Maini AA. The fate and lifespan of human monocyte subsets in steady state and systemic inflammation. J Exp Med. (2017) 214:1913–23. 10.1084/jem.20170355 28606987 PMC5502436

[20] Christ A Günther P Lauterbach MAR Duewell P Biswas D Pelka K. Western diet triggers NLRP3-dependent innate immune reprogramming. Cell. (2018) 172:162–75.e14. 10.1016/j.cell.2017.12.013 29328911 PMC6324559

[21] Nagareddy PR Kraakman M Masters SL Stirzaker RA Gorman DJ Grant RW. Adipose tissue macrophages promote myelopoiesis and monocytosis in obesity. Cell Metab. (2014) 19:821–35. 10.1016/j.cmet.2014.03.029 24807222 PMC4048939

[22] Hamers AAJ Dinh HQ Thomas GD Marcovecchio P Blatchley A Nakao CS. Human monocyte heterogeneity as revealed by high-dimensional mass cytometry. Arterioscler Thromb Vasc Biol. (2019) 39:25–36 10.1161/ATVBAHA.118.311022 30580568 PMC6697379

[23] Banchereau J Steinman RM. Dendritic cells and the control of immunity. Nature. (1998) 392:245–52. 10.1038/32588 9521319

[24] Steinman RM Cohn ZA. Identification of a novel cell type in peripheral lymphoid organs of mice. I. Morphology, quantitation, tissue distribution. J Exp Med. (1973) 137:1142–62. 10.1084/jem.137.5.1142 4573839 PMC2139237

[25] Steinman RM Witmer MD. Lymphoid dendritic cells are potent stimulators of the primary mixed leukocyte reaction in mice. Proc Natl Acad Sci USA. (1978) 75:5132–6. 10.1073/pnas.75.10.5132 154105 PMC336278

[26] Auffray C Sieweke MH Geissmann F. Blood monocytes: development, heterogeneity, and relationship with dendritic cells. Annu Rev Immunol. (2009) 27:669–92. 10.1146/annurev.immunol.021908.132557 19132917

[27] Onai N Kurabayashi K Hosoi-Amaike M Toyama-Sorimachi N Matsushima K Inaba K. A clonogenic progenitor with prominent plasmacytoid dendritic cell developmental potential. Immunity. (2013) 38:943–57. 10.1016/j.immuni.2013.04.006 23623382

[28] Naik SH Sathe P Park H-Y Metcalf D Proietto AI Dakic A. Development of plasmacytoid and conventional dendritic cell subtypes from single precursor cells derived *in vitro* and *in vivo*. Nat Immunol. (2007) 8:1217–26. 10.1038/ni1522 17922015

[29] Schlitzer A Sivakamasundari V Chen J Sumatoh Bin HR Schreuder J Lum J. Identification of cDC1- and cDC2-committed DC progenitors reveals early lineage priming at the common DC progenitor stage in the bone marrow. Nat Immunol. (2015) 16:718–28. 10.1038/ni.3200 26054720

[30] Breton G Zheng S Valieris R Tojal da Silva I Satija R Nussenzweig MC Human dendritic cells (DCs) are derived from distinct circulating precursors that are precommitted to become CD1c+ or CD141+ DCs. J Exp Med. (2016) 213:2861–70. 10.1084/jem.20161135 27864467 PMC5154947

[31] Rodrigues PFF Alberti-Servera L Eremin A Grajales-Reyes GEE Ivanek R Tussiwand R. Distinct progenitor lineages contribute to the heterogeneity of plasmacytoid dendritic cells. Nat Immunol. (2018) 19:711–22. 10.1038/s41590-018-0136-9 29925996 PMC7614340

[32] Murray EGD Webb RA Swann MBR A disease of rabbits characterised by a large mononuclear leucocytosis, caused by a hitherto undescribed bacillusBacterium monocytogenes (n.sp.). J Pathol Bacteriol. (1926) 29:407–39. 10.1002/path.1700290409

[33] Metchnikoff E Ueber den Kampf der Zellen gegen Erysipelkokken. Ein Beitrag zur Phagocytenlehre. Arch für Pathol Anat und Physiol und für Klin Med. (1887) 107:209–49. 10.1007/BF01926053

[34] Figdor C Bont W Touw I de Roos J Roosnek E de Vries J. Isolation of functionally different human monocytes by counterflow centrifugation elutriation. Blood. (1982) 60:46–53. 7082846

[35] Akiyama Y Miller PJ Thurman GB Neubauer RH Oliver C Favilla T. Characterization of a human blood monocyte subset with low peroxidase activity. J Clin Invest. (1983) 72:1093–105. 10.1172/JCI111034 6193141 PMC1129277

[36] Akiyama Y Stevenson GW Schlick E Matsushima K Miller PJ Stevenson HC. Differential ability of human blood monocyte subsets to release various cytokines. J Leukoc Biol. (1985) 37:519–30. 10.1002/jlb.37.5.519 2984302

[37] Elias J Chien P Gustilo K Schreiber A. Differential interleukin-1 elaboration by density-defined human monocyte subpopulations. Blood. (1985) 66:298–301. 3874661

[38] Ebert RH Florey HW THE extravascular development of the monocyte observed *in vivo*. Br J Exp Pathol. (1939) 20:342–56.

[39] Metchnikoff E Leçons sur la Pathologie Comparée de L'inflammation : Faites à l'Institut Pasteur en Avril et mai 1891. Paris: G Masson (1892).

[40] van Furth R Cohn ZA. The origin and kinetics of mononuclear phagocytes. J Exp Med. (1968) 128:415–35. 10.1084/jem.128.3.415 5666958 PMC2138527

[41] Adams DO Edelson PJ Koren HS Methods for Studying Mononuclear Phagocytes. New York, NY: Academic Press (1981). 1023 p.

[42] Steinman RM. The Dendritic cell system and its role in immunogenicity. Annu Rev Immunol. (1991) 9:271–96. 10.1146/annurev.iy.09.040191.001415 1910679

[43] Takahashi K Yamamura F Naito M. Differentiation, maturation, and proliferation of macrophages in the mouse yolk sac: a light-microscopic, enzyme-cytochemical, immunohistochemical, and ultrastructural study. J Leukoc Biol. (1989) 45:87–96. 10.1002/jlb.45.2.87 2536795

[44] Ginhoux F Greter M Leboeuf M Nandi S See P Gokhan S. Fate mapping analysis reveals that adult microglia derive from primitive macrophages. Science. (2010) 330:841–5. 10.1126/science.1194637 20966214 PMC3719181

[45] Schulz C Perdiguero EG Chorro L Szabo-Rogers H Cagnard N Kierdorf K. A lineage of myeloid cells independent of myb and hematopoietic stem cells. Science. (2012) 336:86–90. 10.1126/science.1219179 22442384

[46] Hashimoto D Chow A Noizat C Teo P Beasley MB Leboeuf M. Tissue-resident macrophages self-maintain locally throughout adult life with minimal contribution from circulating monocytes. Immunity. (2013) 38:792–804. 10.1016/j.immuni.2013.04.004 23601688 PMC3853406

[47] Mass E Ballesteros I Farlik M Halbritter F Günther P Crozet L. Specification of tissue-resident macrophages during organogenesis. Science. (2016) 353:aaf4238. 10.1126/science.aaf4238 27492475 PMC5066309

[48] Ginhoux F Schultze JL Murray PJ Ochando J Biswas SK. New insights into the multidimensional concept of macrophage ontogeny, activation and function. Nat Immunol. (2016) 17:34–40. 10.1038/ni.3324 26681460

[49] Yasaka T Mantich NM Boxer LA Baehner RL. Functions of human monocyte and lymphocyte subsets obtained by countercurrent centrifugal elutriation: differing functional capacities of human monocyte subsets. J Immunol. (1981) 127:1515–8. 6268707

[50] Passlick B Flieger D Ziegler-Heitbrock HW. Identification and characterization of a novel monocyte subpopulation in human peripheral blood. Blood. (1989) 74:2527–34. 2478233

[51] Ziegler-Heitbrock HWL Ulevitch RJ. CD14: cell surface receptor and differentiation marker. Immunol Today. (1993) 14:121–5. 10.1016/0167-5699(93)90212-4 7682078

[52] Grage-Griebenow E Zawatzky R Kahlert H Brade L Flad H-D Ernst M. Identification of a novel dendritic cell-like subset of CD64+/CD16+ blood monocytes. Eur J Immunol. (2001) 31:48–56. 10.1002/1521-4141(200101)31:1<48::AID-IMMU48>3.0.CO;2-5 11169437

[53] Grage-Griebenow E Flad HD Ernst M. Heterogeneity of human peripheral blood monocyte subsets. J Leukoc Biol. (2001) 69:11–20. 10.1189/jlb.69.1.11 11200054

[54] Maurer D Fiebiger E Reininger B Wolff-Winiski B Jouvin MH Kilgus O. Expression of functional high affinity immunoglobulin E receptors (Fc epsilon RI) on monocytes of atopic individuals. J Exp Med. (1994) 179:745–50. 10.1084/jem.179.2.745 8294882 PMC2191351

[55] Crawford K Gabuzda D Pantazopoulos V Xu J Clement C Reinherz E. Cells monocytes are dendritic + circulating CD2. J Immunol References. (1999) 163:5920–8. 10570278

[56] Cheng YX Foster B Holland SM Klion AD Nutman TB Casale TB CD2 identifies a monocyte subpopulation with immunoglobulin E-dependent, high-level expression of Fc?RI. Clin Exp Allergy. (2006) 36:1436–45. 10.1111/j.1365-2222.2006.02578.x 17083354 PMC1661841

[57] Reizis B Bunin A Ghosh HS Lewis KL Sisirak V. Plasmacytoid dendritic cells: recent progress and open questions. Annu Rev Immunol. (2011) 29:163–83. 10.1146/annurev-immunol-031210-101345 21219184 PMC4160806

[58] Rönnblomo L Ramstedt U Alm GV Properties of human natural interferon-producing cells stimulated by tumor cell lines. Eur J Immunol. (1983) 13:471–6. 10.1002/eji.1830130608 6574913

[59] Chehimi J Starr SE Kawashima H Miller DS Trinchieri G Perussia B. Dendritic cells and IFN-alpha-producing cells are two functionally distinct non-B, non-monocytic HLA-DR+ cell subsets in human peripheral blood. Immunology. (1989) 68:486–90. 2532619 PMC1385535

[60] Desmet FFW-PKRV van den O The American journal of surgical pathology. Am J Surg Pathol. (1990) 14:101–12. 10.1097/00000478-199002000-00001 2301697

[61] Grouard G Rissoan M-C Filgueira L Durand I Banchereau J Liu Y-J. The enigmatic plasmacytoid T cells develop into dendritic cells with Interleukin (IL)-3 and CD40-ligand. J Exp Med. (1997) 185:1101. 10.1084/jem.185.6.1101 9091583 PMC2196227

[62] Siegal FPP Kadowaki N Shodell M Fitzgerald-Bocarsly PAA Shah K Ho S. The nature of the principal type 1 interferon-producing cells in human blood. Science. (1999) 284:1835–7. 10.1126/science.284.5421.1835 10364556

[63] Cella M Jarrossay D Facchetti F Alebardi O Nakajima H Lanzavecchia A. Plasmacytoid monocytes migrate to inflamed lymph nodes and produce large amounts of type I interferon. Nat Med. (1999) 5:919–23. 10.1038/11360 10426316

[64] Dzionek A Fuchs A Schmidt P Cremer S Zysk M Miltenyi S. BDCA-2, BDCA-3, and BDCA-4: three markers for distinct subsets of dendritic cells in human peripheral blood. J Immunol. (2000) 165:6037–46. 10.4049/jimmunol.165.11.6037 11086035

[65] MacDonald KPAPA Munster DJJ Clark GJJ Dzionek A Schmitz J Hart DNJNJ. Characterization of human blood dendritic cell subsets. Blood. (2002) 100:4512–20. 10.1182/blood-2001-11-0097 12393628

[66] O'Doherty U Peng M Gezelter S Swiggard WJ Betjes M Bhardwaj N. Human blood contains two subsets of dendritic cells, one immunologically mature and the other immature. Immunology. (1994) 82:487–93. 7525461 PMC1414873

[67] Thomas R Davis LSS Lipsky PEE. Isolation and characterization of human peripheral blood dendritic cells. J Immunol. (1993) 150:821–34. 7678623

[68] Schlitzer A McGovern N Ginhoux F. Dendritic cells and monocyte-derived cells: Two complementary and integrated functional systems. Semin Cell Dev Biol. (2015) 41:9–22. 10.1016/j.semcdb.2015.03.011 25957517

[69] Schlitzer A Zhang W Song M Ma X. Recent advances in understanding dendritic cell development, classification, and phenotype. F1000Research. (2018) 7:1558. 10.12688/f1000research.14793.1 30345015 PMC6173131

[70] Vremec D Shortman K. What's in a name? Some early and current issues in dendritic cell nomenclature. Front Immunol. (2015) 6:267. 10.3389/fimmu.2015.00267 26074925 PMC4448511

[71] Merad M Sathe P Helft J Miller J Mortha A. The dendritic cell lineage: ontogeny and function of dendritic cells and their subsets in the steady state and the inflamed setting. Annu Rev Immunol. (2013) 31:563–604. 10.1146/annurev-immunol-020711-074950 23516985 PMC3853342

[72] Bassler K Schulte-Schrepping J Warnat-Herresthal S Aschenbrenner AC Schultze JL. The myeloid cell compartment—cell by cell. Annu Rev Immunol. (2019) 37:269–93. 10.1146/annurev-immunol-042718-041728 30649988

[73] Guilliams M Ginhoux F Jakubzick C Naik SH Onai N Schraml BU. Dendritic cells, monocytes and macrophages: a unified nomenclature based on ontogeny. Nat Rev Immunol. (2014) 14:571–8. 10.1038/nri3712 25033907 PMC4638219

[74] Heidkamp GF Sander J Lehmann CHK Heger L Eissing N Baranska A Human lymphoid organ dendritic cell identity is predominantly dictated by ontogeny, not tissue microenvironment. Sci Immunol. (2016) 1:eaai7677 10.1126/sciimmunol.aai7677 28783692

[75] Bachem A Güttler S Hartung E Ebstein F Schaefer M Tannert A. Superior antigen cross-presentation and XCR1 expression define human CD11c+CD141+ cells as homologues of mouse CD8+ dendritic cells. J Exp Med. (2010) 207:1273–81. 10.1084/jem.20100348 20479115 PMC2882837

[76] Crozat K Guiton R Contreras V Feuillet V Dutertre C-A Ventre E. The XC chemokine receptor 1 is a conserved selective marker of mammalian cells homologous to mouse CD8alpha+ dendritic cells. J Exp Med. (2010) 207:1283–92. 10.1084/jem.20100223 20479118 PMC2882835

[77] Caminschi I Proietto AII Ahmet F Kitsoulis S Teh JSS Lo JCYCY. The dendritic cell subtype-restricted C-type lectin Clec9A is a target for vaccine enhancement. Blood. (2008) 112:3264–73. 10.1182/blood-2008-05-155176 18669894 PMC2569177

[78] Huysamen C Willment JA Dennehy KM Brown GD. CLEC9A Is a novel activation C-type lectin-like receptor expressed on BDCA3(+) dendritic cells and a subset of monocytes. J Biol Chem. (2008) 283:16693–701. 10.1074/jbc.M709923200 18408006 PMC2562446

[79] Sancho D Joffre OPP Keller AMM Rogers NCC Martínez D Hernanz-Falcón P. Identification of a dendritic cell receptor that couples sensing of necrosis to immunity. Nature. (2009) 458:899–903. 10.1038/nature07750 19219027 PMC2671489

[80] Haniffa M Shin A Bigley V McGovern N Teo P See P. Human tissues contain CD141hi cross-presenting dendritic cells with functional homology to mouse CD103+ nonlymphoid dendritic cells. Immunity. (2012) 37:60–73. 10.1016/j.immuni.2012.04.012 22795876 PMC3476529

[81] Sittig SPP Bakdash G Weiden J Sköld AEE Tel J Figdor CGG. A comparative study of the T cell stimulatory and polarizing capacity of human primary blood dendritic cell subsets. Mediators Inflamm. (2016) 2016:1–11. 10.1155/2016/3605643 27057096 PMC4761397

[82] Schulz O Diebold SSS Chen M Näslund TII Nolte MAA Alexopoulou L. Toll-like receptor 3 promotes cross-priming to virus-infected cells. Nature. (2005) 433:887–92. 10.1038/nature03326 15711573

[83] Schlitzer A Ginhoux F. Organization of the mouse and human DC network. Curr Opin Immunol. (2014) 26:90–9. 10.1016/j.coi.2013.11.002 24556405

[84] Ito T Inaba M Inaba K Toki J Sogo S Iguchi T. A CD1a+/CD11c+ subset of human blood dendritic cells is a direct precursor of langerhans cells. J Immunol. (1999) 163:1409–19. 10415041

[85] Ginhoux F Guilliams M. Tissue-resident macrophage ontogeny and homeostasis. Immunity. (2016) 44:439–49. 10.1016/j.immuni.2016.02.024 26982352

[86] Kawamura S Ohteki T. Monopoiesis in humans and mice. Int Immunol. (2018) 30:503–9. 10.1093/intimm/dxy063 30247712

[87] Ito T Kanzler H Duramad O Cao W Liu Y-J. Specialization, kinetics, and repertoire of type 1 interferon responses by human plasmacytoid predendritic cells. Blood. (2006) 107:2423–31. 10.1182/blood-2005-07-2709 16293610

[88] Dalod M Salazar-Mather TP Malmgaard L Lewis C Asselin-Paturel C Brière F. Interferon α/β and interleukin 12 responses to viral infections. J Exp Med. (2002) 195:517–28. 10.1084/jem.20011672 11854364 PMC2193614

[89] Quinn ZA Baranov VI Tanner SD Wrana JL Simultaneous determination of proteins using an element-tagged immunoassay coupled with ICP-MS detection. J Anal At Spectrom. (2002) 17:892–6. 10.1039/b202306g

[90] Baranov VI Quinn ZA Bandura DR Tanner SD The potential for elemental analysis in biotechnology. J Anal At Spectrom. (2002) 17:1148–52. 10.1039/B201494G

[91] Ornatsky O Baranov VI Bandura DR Tanner SD Dick J. Multiple cellular antigen detection by ICP-MS. J Immunol Methods. (2006) 308:68–76. 10.1016/j.jim.2005.09.020 16336974

[92] Lou X Zhang G Herrera I Kinach R Ornatsky O Baranov V. Polymer-based elemental tags for sensitive bioassays. Angew Chemie Int Ed. (2007) 46:6111–4. 10.1002/anie.200700796 17533637 PMC2504858

[93] Ornatsky O Bandura D Baranov V Nitz M Winnik MA Tanner S. Highly multiparametric analysis by mass cytometry. J Immunol Methods. (2010) 361:1–20. 10.1016/j.jim.2010.07.002 20655312

[94] Bendall SC Simonds EF Qiu P Amir E-a D Krutzik PO Finck R. Single-cell mass cytometry of differential immune and drug responses across a human hematopoietic continuum. Science. (2011) 332:687–96. 10.1126/science.1198704 21551058 PMC3273988

[95] Bendall SCC Davis KLL Amir EDD Tadmor MDD Simonds EFF Chen TJJ. Single-cell trajectory detection uncovers progression and regulatory coordination in human B cell development. Cell. (2014) 157:714–25. 10.1016/j.cell.2014.04.005 24766814 PMC4045247

[96] Newell EW Sigal N Bendall SC Nolan GP Davis MM. Cytometry by time-of-flight shows combinatorial cytokine expression and virus-specific cell niches within a continuum of CD8+ T cell phenotypes. Immunity. (2012) 36:142–52. 10.1016/j.immuni.2012.01.002 22265676 PMC3752833

[97] Newell EW Sigal N Nair N Kidd BA Greenberg HB Davis MM. Combinatorial tetramer staining and mass cytometry analysis facilitate T-cell epitope mapping and characterization. Nat Biotechnol. (2013) 31:623–9. 10.1038/nbt.2593 23748502 PMC3796952

[98] Nagalakshmi U Wang Z Waern K Shou C Raha D Gerstein M. The transcriptional landscape of the yeast genome defined by RNA sequencing. Science. (2008) 320:1344–9. 10.1126/science.1158441 18451266 PMC2951732

[99] Wang Z Gerstein M Snyder M. RNA-Seq: a revolutionary tool for transcriptomics. Nat Rev Genet. (2009) 10:57–63. 10.1038/nrg2484 19015660 PMC2949280

[100] Xue J Schmidt SV Sander J Draffehn A Krebs W Quester I. Transcriptome-based network analysis reveals a spectrum model of human macrophage activation. Immunity. (2014) 40:274–88. 10.1016/j.immuni.2014.01.006 24530056 PMC3991396

[101] Schmidt SV Krebs W Ulas T Xue J Baßler K Günther P. The transcriptional regulator network of human inflammatory macrophages is defined by open chromatin. Cell Res. (2016) 26:151–70. 10.1038/cr.2016.1 26729620 PMC4746609

[102] Sander J Schmidt SV Cirovic B McGovern N Papantonopoulou O Hardt A-L. Cellular differentiation of human monocytes is regulated by time-dependent interleukin-4 signaling and the transcriptional regulator NCOR2. Immunity. (2017) 47:1051–66.e12. 10.1016/j.immuni.2017.11.024 29262348 PMC5772172

[103] Lindstedt M Lundberg K Borrebaeck CAK. Gene family clustering identifies functionally associated subsets of human *in vivo* blood and tonsillar dendritic cells. J Immunol. (2005) 175:4839–46. 10.4049/jimmunol.175.8.4839 16210585

[104] Robbins SH Walzer T Dembélé D Thibault C Defays A Bessou G. Novel insights into the relationships between dendritic cell subsets in human and mouse revealed by genome-wide expression profiling. Genome Biol. (2008) 9:R17. 10.1186/gb-2008-9-1-r17 18218067 PMC2395256

[105] Crozat K Guiton R Guilliams M Henri S Baranek T Schwartz-Cornil I. Comparative genomics as a tool to reveal functional equivalences between human and mouse dendritic cell subsets. Immunol Rev. (2010) 234:177–98. 10.1111/j.0105-2896.2009.00868.x 20193019

[106] Lavin Y Winter D Blecher-Gonen R David E Keren-Shaul H Merad M. Tissue-resident macrophage enhancer landscapes are shaped by the local microenvironment. Cell. (2014) 159:1312–26. 10.1016/j.cell.2014.11.018 25480296 PMC4437213

[107] Gosselin D Link VMM Romanoski CEE Fonseca GJJ Eichenfield DZZ Spann NJJ. Environment drives selection and function of enhancers controlling tissue-specific macrophage identities. Cell. (2014) 159:1327–40. 10.1016/j.cell.2014.11.023 25480297 PMC4364385

[108] Vu Manh T-P Bertho N Hosmalin A Schwartz-Cornil I Dalod M. Investigating evolutionary conservation of dendritic cell subset identity and functions. Front Immunol. (2015) 6:260. 10.3389/fimmu.2015.00260 26082777 PMC4451681

[109] Ingersoll MAA Spanbroek R Lottaz C Gautier ELL Frankenberger M Hoffmann R. Comparison of gene expression profiles between human and mouse monocyte subsets. Blood. (2010) 115:e10–9. 10.1182/blood-2009-07-235028 19965649 PMC2810986

[110] Cros J Cagnard N Woollard K Patey N Zhang S-Y Senechal B. Human CD14dim monocytes patrol and sense nucleic acids and viruses via TLR7 and TLR8 receptors. Immunity. (2010) 33:375–86. 10.1016/j.immuni.2010.08.012 20832340 PMC3063338

[111] Wong KLL Tai JJ-YJ-Y Wong W-C Han H Sem X Yeap W-H. Gene expression profiling reveals the defining features of the classical, intermediate, and nonclassical human monocyte subsets. Blood. (2011) 118:e16–31. 10.1182/blood-2010-12-326355 21653326

[112] Frankenberger M Hofer TPJPJ Marei A Dayyani F Schewe S Strasser C. Transcript profiling of CD16-positive monocytes reveals a unique molecular fingerprint. Eur J Immunol. (2012) 42:957–74. 10.1002/eji.201141907 22531920

[113] Vä L Scheele C Pedersen K Nielsen J Proteome-and transcriptome-driven reconstruction of the human myocyte metabolic network and its use for identification of markers for diabetes accession numbers. Cell Rep. (2015) 11:921–33. 10.1016/j.celrep.2015.04.010 25937284

[114] Maier T Güell M Serrano L. Correlation of mRNA and protein in complex biological samples. FEBS Lett. (2009) 583:3966–73. 10.1016/j.febslet.2009.10.036 19850042

[115] Narita T Weinert BTT Choudhary C Functions and mechanisms of non-histone protein acetylation. Nat Rev Mol Cell Biol. (2019) 20:156–74. 10.1038/s41580-018-0081-3 30467427

[116] Wang Y-C Peterson SEE Loring JFF. Protein post-translational modifications and regulation of pluripotency in human stem cells. Cell Res. (2014) 24:143–60. 10.1038/cr.2013.151 24217768 PMC3915910

[117] Spitzer MH Nolan GP. Mass cytometry: single cells, many features. Cell. (2016) 165:780–91. 10.1016/j.cell.2016.04.019 27153492 PMC4860251

[118] Bodenmiller B Zunder ER Finck R Chen TJ Savig ES Bruggner RV. Multiplexed mass cytometry profiling of cellular states perturbed by small-molecule regulators. Nat Biotechnol. (2012) 30:858–67. 10.1038/nbt.2317 22902532 PMC3627543

[119] Schäkel K Kannagi R Kniep B Goto Y Mitsuoka C Zwirner J. 6-Sulfo LacNAc, a novel carbohydrate modification of PSGL-1, defines an inflammatory type of human dendritic cells. Immunity. (2002) 17:289–301. 10.1016/S1074-7613(02)00393-X 12354382

[120] Schäkel K von Kietzell M Hänsel A Ebling A Schulze L Haase M. Human 6-sulfo LacNAc-expressing dendritic cells are principal producers of early interleukin-12 and are controlled by erythrocytes. Immunity. (2006) 24:767–77. 10.1016/j.immuni.2006.03.020 16782032

[121] Micheletti A Finotti G Calzetti F Lonardi S Zoratti E Bugatti M slan/M-DC8_+_ cells constitute a distinct subset of dendritic cells in human tonsils. Oncotarget. (2016) 7:161–75. 10.18632/oncotarget.12418 26695549 PMC4807990

[122] Hofer TPP Zawada AMM Frankenberger M Skokann K Satzl AAA Gesierich W. slan-defined subsets of CD16-positive monocytes: impact of granulomatous inflammation and M-CSF receptor mutation. Blood. (2015) 126:2601–10. 10.1182/blood-2015-06-651331 26443621

[123] Siedlarl M Frankenberger M Ziegler-Heitbrock LHWHW Belge K-U The M-DC8-positive leukocytes are a subpopulation of the CD14+CD16+monocytes. Immunobiology. (2000) 202:11–7. 10.1016/S0171-2985(00)80047-9 10879684

[124] Roussel M Ferrell PB Greenplate AR Lhomme F Le Gallou S Diggins KE. Mass cytometry deep phenotyping of human mononuclear phagocytes and myeloid-derived suppressor cells from human blood and bone marrow. J Leukoc Biol. (2017) 102:437–47. 10.1189/jlb.5MA1116-457R 28400539 PMC6608074

[125] Günther P Cirovic B Baßler K Händler K Becker M Dutertre CAA A rule-based data-informed cellular consensus map of the human mononuclear phagocyte cell space. bioRxiv [Preprint]. (2019) 658179 10.1101/658179

[126] Thomas GD Hamers AAJ Nakao C Marcovecchio P Taylor AM McSkimming C. Human blood monocyte subsets. Arterioscler Thromb Vasc Biol. (2017) 37:1548–58. 10.1161/ATVBAHA.117.309145 28596372 PMC5828170

[127] Amir ED Davis KL Tadmor MD Simonds EF Levine JH Bendall SC viSNE enables visualization of high dimensional single-cell data and reveals phenotypic heterogeneity of leukemia. Nat Biotechnol. (2013) 31:545–52. 10.1038/nbt.2594 23685480 PMC4076922

[128] Samusik N Good Z Spitzer MH Davis KL Nolan GP. Automated mapping of phenotype space with single-cell data. Nat Methods. (2016) 13:493–6. 10.1038/nmeth.3863 27183440 PMC4896314

[129] Johansson MWW. Eosinophil activation status in separate compartments and association with asthma. Front Med. (2017) 4:75. 10.3389/fmed.2017.00075 28660189 PMC5466952

[130] Han X Jorgensen JLL Brahmandam A Schlette E Huh YOO Shi Y. Immunophenotypic study of basophils by multiparameter flow cytometry. Arch Pathol Lab Med. (2008) 132:813–9. 10.1043/1543-2165(2008)132[813:ISOBBM]2.0.CO;2 18466030

[131] Mukai K Gaudenzio N Gupta S Vivanco N Bendall SCC Maecker HTT. Assessing basophil activation by using flow cytometry and mass cytometry in blood stored 24 hours before analysis. J Allergy Clin Immunol. (2017) 139:889–99.e11. 10.1016/j.jaci.2016.04.060 27527263 PMC5237629

[132] Alcántara-Hernández M Leylek R Wagar LE Engleman EG Keler T Marinkovich MP. High-dimensional phenotypic mapping of human dendritic cells reveals interindividual variation and tissue specialization. Immunity. (2017) 47:1037–50.e6. 10.1016/j.immuni.2017.11.001 29221729 PMC5738280

[133] Tang F Barbacioru C Wang Y Nordman E Lee C Xu N. mRNA-Seq whole-transcriptome analysis of a single cell. Nat Methods. (2009) 6:377–82. 10.1038/nmeth.1315 19349980

[134] Tang F Barbacioru C Nordman E Li B Xu N Bashkirov VI. RNA-Seq analysis to capture the transcriptome landscape of a single cell. Nat Protoc. (2010) 5:516–35. 10.1038/nprot.2009.236 20203668 PMC3847604

[135] Aevermann BD Novotny M Bakken T Miller JA Diehl AD Osumi-Sutherland D. Cell type discovery using single-cell transcriptomics: implications for ontological representation. Hum Mol Genet. (2018) 27:R40–7. 10.1093/hmg/ddy100 29590361 PMC5946857

[136] Tanay A Regev A. Scaling single-cell genomics from phenomenology to mechanism. Nature. (2017) 541:331–8. 10.1038/nature21350 28102262 PMC5438464

[137] Junker JP Van Oudenaarden A. Every cell is special: genome-wide studies add a new dimension to single-cell biology. Cell. (2014) 157:8–11. 10.1016/j.cell.2014.02.010 24679522

[138] Shapiro E Biezuner T Linnarsson S. Single-cell sequencing-based technologies will revolutionize whole-organism science. Nat Rev Genet. (2013) 14:618–30. 10.1038/nrg3542 23897237

[139] The Tabula Muris Consortium Single-cell transcriptomics of 20 mouse organs creates a Tabula Muris. Nature. (2018) 562:367–72. 10.1038/s41586-018-0590-4 30283141 PMC6642641

[140] Eberwine J Sul J-Y Bartfai T Kim J. The promise of single-cell sequencing. Nat Methods. (2013) 11:25–7. 10.1038/nmeth.2769 24524134

[141] Papalexi E Satija R. Single-cell RNA sequencing to explore immune cell heterogeneity. Nat Rev Immunol. (2017) 18:35–45. 10.1038/nri.2017.76 28787399

[142] Cao J Packer JSS Ramani V Cusanovich DAA Huynh C Daza R. Comprehensive single-cell transcriptional profiling of a multicellular organism. Science. (2017) 357:661–7. 10.1126/science.aam8940 28818938 PMC5894354

[143] Villani A-C Satija R Reynolds G Sarkizova S Shekhar K Fletcher J. Single-cell RNA-seq reveals new types of human blood dendritic cells, monocytes, and progenitors. Science. (2017) 356:eaah4573. 10.1126/science.aah4573 28428369 PMC5775029

[144] Collin M Bigley V. Human dendritic cell subsets: an update. Immunology. (2018) 154:3–20. 10.1111/imm.12888 29313948 PMC5904714

[145] Laurenti E Göttgens B. From haematopoietic stem cells to complex differentiation landscapes. Nature. (2018) 553:418–26. 10.1038/nature25022 29364285 PMC6555401

[146] Helft J Anjos-Afonso F van der Veen AG Chakravarty P Bonnet D Reise Sousa C. Dendritic cell lineage potential in human early hematopoietic progenitors. Cell Rep. (2017) 20:529–37. 10.1016/j.celrep.2017.06.075 28723558 PMC5529209

[147] Kolodziejczyk AA Kim JK Tsang JCH Ilicic T Henriksson J Natarajan KN. Single cell RNA-sequencing of pluripotent states unlocks modular transcriptional variation. Cell Stem Cell. (2015) 17:471–85. 10.1016/j.stem.2015.09.011 26431182 PMC4595712

[148] Buettner F Natarajan KN Casale FP Proserpio V Scialdone A Theis FJ. Computational analysis of cell-to-cell heterogeneity in single-cell RNA-sequencing data reveals hidden subpopulations of cells. Nat Biotechnol. (2015) 33:155–60. 10.1038/nbt.3102 25599176

[149] Wang J Huang M Torre E Dueck H Shaffer S Murray J. Gene expression distribution deconvolution in single-cell RNA sequencing. Proc Natl Acad Sci USA. (2018) 115:E6437–46. 10.1073/pnas.1721085115 29946020 PMC6048536

[150] Stegle O Teichmann SA Marioni JC. Computational and analytical challenges in single-cell transcriptomics. Nat Rev Genet. (2015) 16:133–45. 10.1038/nrg3833 25628217

[151] Hoppe PS Schwarzfischer M Loeffler D Kokkaliaris KD Hilsenbeck O Moritz N Early myeloid lineage choice is not initiated by random PU.1 to GATA1 protein ratios. Nature. (2016) 535:299–302. 10.1038/nature18320 27411635

[152] Giladi A Paul F Herzog Y Lubling Y Weiner A Yofe I. Single-cell characterization of haematopoietic progenitors and their trajectories in homeostasis and perturbed haematopoiesis. Nat Cell Biol. (2018) 20:836–46. 10.1038/s41556-018-0121-4 29915358

[153] Franziska Paul A Arkin ara Giladi A Torben Porse B Tanay A Amit I. Transcriptional heterogeneity and lineage commitment in myeloid progenitors. Cell. (2015);163:1663–77. 10.1016/j.cell.2015.11.013 26627738

[154] Shahi P Kim SCC Haliburton JRR Gartner ZJJ Abate ARR. Abseq: ultrahigh-throughput single cell protein profiling with droplet microfluidic barcoding. Sci Rep. (2017) 7:44447. 10.1038/srep44447 28290550 PMC5349531

[155] Stoeckius M Hafemeister C Stephenson W Houck-Loomis B Chattopadhyay PKK Swerdlow H. Simultaneous epitope and transcriptome measurement in single cells. Nat Methods. (2017) Sep;14:865–8. 10.1038/nmeth.4380 28759029 PMC5669064

[156] Engel P Boumsell L Balderas R Bensussan A Gattei V Horejsi V. CD nomenclature 2015: human leukocyte differentiation antigen workshops as a driving force in immunology. J Immunol. (2015) 195:4555–63. 10.4049/jimmunol.1502033 26546687

[157] Regev A Teichmann SA Lander ES Amit I Benoist C Birney E The human cell atlas meeting participants. bioRxiv [Preprint]. (2017). 10.1101/121202

[158] Wagner A Regev A Yosef N. Revealing the vectors of cellular identity with single-cell genomics. Nat Biotechnol. (2016) 34:1145–60. 10.1038/nbt.3711 27824854 PMC5465644

[159] Aran D Looney APP Liu L Wu E Fong V Hsu A. Reference-based analysis of lung single-cell sequencing reveals a transitional profibrotic macrophage. Nat Immunol. (2019) 20:163–72. 10.1038/s41590-018-0276-y 30643263 PMC6340744

[160] Hou R Denisenko E Forrest ARR. scMatch: a single-cell gene expression profile annotation tool using reference datasets. Kelso J, editor. Bioinformatics. (2019) btz292. 10.1093/bioinformatics/btz292 PMC6853649 31028376

[161] Warnat-Herresthal S Perrakis K Taschler B Becker M Seep L Baßler K Diagnostic value of blood gene expression-based classifiers as exemplified for acute myeloid leukemia. bioRxiv [Preprint]. (2018) 382143 10.1101/382143

[162] Angra S Ahuja S Machine learning and its applications: a review. In: 2017 International Conference on Big Data Analytics and Computational Intelligence (ICBDAC). Chirala: IEEE (2017). p. 57–60. 10.1109/ICBDACI.2017.8070809

[163] Guilliams M Henri S Tamoutounour S Ardouin L Schwartz-Cornil I Dalod M. From skin dendritic cells to a simplified classification of human and mouse dendritic cell subsets. Eur J Immunol. (2010) 40:2089–94. 10.1002/eji.201040498 20853491

[164] Ginhoux F Jung S. Monocytes and macrophages: developmental pathways and tissue homeostasis. Nat Rev Immunol. (2014) 14:392–404. 10.1038/nri3671 24854589

[165] Arendt D. The evolution of cell types in animals: emerging principles from molecular studies. Nat Rev Genet. (2008) 9:868–82. 10.1038/nrg2416 18927580

[166] Trapnell C. Defining cell types and states with single-cell genomics. Genome Res. (2015) 25:1491–8. 10.1101/gr.190595.115 26430159 PMC4579334

[167] Arendt D Musser JM Baker CVH Bergman A Cepko C Erwin DH. The origin and evolution of cell types. Nat Rev Genet. (2016) 17:744–57. 10.1038/nrg.2016.127 27818507

[168] Marioni JC Arendt D. How single-cell genomics is changing evolutionary and developmental biology. Annu Rev Cell Dev Biol. (2017) 33:537–53. 10.1146/annurev-cellbio-100616-060818 28813177

